# Downstream processing of bioproducts

**DOI:** 10.1002/elsc.202000109

**Published:** 2021-10-15

**Authors:** Sonja Berensmeier

**Affiliations:** ^1^ Bioseparation Engineering Group Technical University Munich Munich Germany

It was my pleasure to organize, together with my colleague Zhi‐Long Xiu, the Special Issue *Downstream processing of bioproducts*, which we divided into two issues due to the large number of articles. Part 1 was published as issue 6/2021; part 2 is the current issue. The great response shows the importance and diversity of the topic.

Depending on the product and the biological production system, a combination of mechanistically different separation methods has to be used, with product quality, purity, and economic viability as the main objectives. Particular challenges based on upstream processing – biotechnological production – include the processing of large volumes of water, the solubility of the products in the aqueous system, and any product inhibition that may require in situ product removal. Other important target parameters are the selectivities and yields of the individual process units, in order to develop the most efficient overall process.

This issue shows new process ideas that have been developed for the separation of whole cells or macromolecules like mRNA and proteins, and also for equally relevant smaller molecules like terpenes, antibiotics, and alcohols, which are very different target molecules for which individual processes have to be developed. Innovative ideas are methods such as magnetic separation and capacitive deionization, and a low‐cost alternative for affinity separation of proteins is also given. Traditionally established extraction‐ and membrane‐separation methods, which are still very good and important bases, essential for multistep downstream processing, are also discussed.

